# Crystallization of SAPO-11 Molecular Sieves Prepared from Silicoaluminophosphate Gels Using Boehmites with Different Properties

**DOI:** 10.3390/gels9020123

**Published:** 2023-02-02

**Authors:** Marat R. Agliullin, Svetlana V. Cherepanova, Zulfiya R. Fayzullina, Dmitry V. Serebrennikov, Leonard M. Khalilov, Tatyana R. Prosochkina, Boris I. Kutepov

**Affiliations:** 1 Ufa Federal Research Centre of the Russian Academy of Sciences (UFRC RAS), Institute of Petrochemistry and Catalysis, 450075 Ufa, Russia; 2 Federal Research Center Boreskov Institute of Catalysis SB RAS, 630090 Novosibirsk, Russia; 3 Faculty of Technology, Ufa State Petroleum Technological University, 450064 Ufa, Russia

**Keywords:** silicoaluminophosphate gels, boehmite, silicoaluminophosphate SAPO-11, nanocrystals, hierarchical zeolites

## Abstract

In this article, we report the results of research the formation of silicoaluminophosphate gels under changing gel aging conditions and the influence of an aluminum source (boehmite), characterized by different properties. The samples of initial gels were characterized by XRF, X-ray diffraction, MAS NMR ^27^Al and ^31^P, and scanning electron microscopy (SEM). Products of crystallization were characterized by XRF, X-ray diffraction, MAS NMR ^27^Al and ^31^P, scanning electron microscopy (SEM), N_2_-physical adsorption, and IR spectroscopy with pyridine adsorption. It has been established that the chemical and phase composition of aging gels and the products of further crystallization is conditioned by the size of the crystals and the porous structure of boehmite. Methods of management the morphology and secondary porous structure of SAPO-11, including the hierarchical porous structure, are proposed based on the use of boehmits characterized by different properties and changing the aging conditions of the initial gels. SAPO—based catalyst with a hierarchical porous structure showed excellent catalytic performance in dimerization of α-methylstyrene with a high degree of conversion and selectivity for linear isomers.

## 1. Introduction

Catalytic systems based on SAPO-n silicoaluminophosphate molecular sieves are increasingly used in various industrial processes in petrochemistry and oil refining [1]. Thus, SAPO-34 containing catalysts are used in the Methanol to Olefins (MTO) process (synthesis of light olefins from methanol) [2], and catalysts containing SAPO-11 are used in the process of hydroisomerization of n-paraffins of diesel fuel and oil fractions [3,4].

The SAPO-n molecular sieves synthesis first reported by Wison et al., (Union Carbide) in 1984 [5]. The structure of SAPO-n is built from AlO_4_, PO_4_ and SiO_4_ tetrahedra linked through common oxygen atoms. SAPO-n are characterized by a wide variety of structures both in terms of pore size (SAPO-11 6.4 × 3.9 Å, SAPO-5 7.4 × 7.4 Å, VPI-5 12.7 × 12.7 Å), and dimensional (1D-SAPO-11 and SAPO-31, 2D -SAPO-35, 3D-SAPO-18) [6].

Bronsted and Lewis acid sites (BAS and LAS, respectively) in SAPO-n molecular sieves are formed as a result of the incorporation—introduction of Si atoms into the aluminophosphate framework during crystallization. Silicon atoms can be included in the aluminophosphate framework by two mechanisms: SM2 or SM2 + SM3 [7]. In the case of the SM2 mechanism, a single insertion of silicon atoms occurs, while in the case of the SM2 + SM3 mechanism, silicate islands of various sizes are formed. BAS in SAPO-n have less strength than in aluminosilicate zeolites. Such acid sites are called “moderate”.

Among the wide variety of SAPO-n structure types, SAPO-11 molecular sieve (AEL structure type) is of particular interest due to its one-dimensional channel structure with 4.0 × 6.5 Å elliptical pores. Bifunctional catalytic systems based on SAPO-11 are the most selective in the hydroisomerization of C_7+_ n-paraffins [8,9]. The high efficiency of catalysts based on SAPO-11 in the isomerization of cyclohexanone oxime to caprolactam [10] and n-butene to isobutylene [11] has been shown. SAPO-11 showed high selectivity in the methylation of toluene to xylenes [12] and naphthalene to dimethylnaphthalenes [13].

At present, certain successes have been achieved in the synthesis of SAPO-11 molecular sieves in controlling the incorporation of silicon atoms into the aluminophosphate framework, in controlling the acidic properties [14,15,16,17,18,19,20,21,22,23] and the crystal morphology [24], as well as in the synthesis of nanosized and hierarchical molecular sieves [8]. However, for these materials, the formation of silicoaluminophosphate gels and the interconnections of their physicochemical properties with the results of their subsequent crystallization are still insufficiently studied. In most works devoted to the synthesis of SAPO-11, it is proposed to use isopropoxide of aluminum or boehmite as a source of aluminum for the preparation of initial gels [25]. It is important to note that boehmite is the preferred raw material for the preparation of silicoaluminophosphate gels due to its availability and lower cost. In addition, during the hydrolysis of aluminum isopropoxide releases isopropyl alcohol, which can create certain difficulties with the subsequent disposal of the alcohol-containing mother liquor. It should be noted that hydrated aluminum oxides (boehmite) can differ significantly in crystal size and porous structure, which is associated not only with the conditions of their synthesis, but also with the technologies for their production [26]. Thus, differences in the properties of boehmite can also have a significant effect on the properties of the formed silicoaluminophosphate gels and the results of their subsequent crystallization, which has not been practically studied.

Answers to the above questions will reveal the mechanism of boehmite selection for the subsequent preparation of silicoaluminophosphate gels and their subsequent crystallization in SAPO-11, as well as develop new ways to control the morphology of molecular sieve crystals and their degree of dispersion.

Carmen M. Lo’pez et al. [27] studied the preparation of SAPO-11 using its hydroxide and isopropoxide as aluminum sources. Aluminum isopropoxide has been shown to produce high phase purity SAPO-11 at lower SDA/Al_2_O_3_ ratios than aluminum hydroxide, suggesting a significant impact of aluminum source reactivity on crystallization results.

M. Alfonzo et al. [28] used boehmite (Catapal B, Disperal) and gibbsite as sources of Al to crystallize SAPO-11. The authors have shown that only the use of Catapal B boehmite makes it possible to obtain SAPO-11 of high phase purity. Crystallization products prepared from Disperal boehmite gels were a mixture of SAPO-11 and tridymite phases, and in the case of gibbsite, the main crystallization product was a mixture of tridymite and cristabolite. The authors attribute the obtained results to the crystallinity of the aluminum source. Boehmite Catapal B, due to the lowest crystallinity, exhibits a higher reactivity, which allows the formation of initial gels of the desired composition.

We have previously shown [29] that by controlling the chemical and phase composition of the initial silicoluminophosphate gels using various sources of aluminum (boehmite, aluminum isopropoxide) or by changing the aging conditions of the initial gels, one can not only obtain SAPO-11 of high phase purity, regardless of the nature source of Al, but also to regulate the crystal morphology and secondary pore structure of molecular sieves.

Thus, the above results indicate the importance of the properties of the aluminum source for the preparation of reactive silicoaluminophosphate gels during the subsequent crystallization of the materials mentioned above. It is important to note that at present there is practically no information in the literature [25] about the crystallization of SAPO-11 molecular sieves using boehmites with different properties. The use of boehmites with different properties for the preparation of silicoaluminophosphate gels can open up new possibilities in the synthesis of SAPO-11 molecular sieves with a given morphology and secondary porous structure. Therefore, in this work, a detailed study of the influence of the properties of various boehmites, as well as the conditions of aging of the initial gels on their chemical and phase composition, as well as their subsequent crystallization into SAPO-11 molecular sieves, was carried out.

## 2. Results and Discussion

Table 1 shows the designations of samples of gels prepared using boehmite, characterized by different properties. The aging temperature of the reaction gels is shown in parentheses. As we can see, all gel samples are a mixture of di-n-propylamine phosphate and unreacted boehmite. The results obtained indicate a weak force between the source of aluminum and phosphoric acid at the initial stage of preparation of such gels.

As noted earlier, the physicochemical properties of the initial silicoaluminophosphate gels can have a significant effect on the results of their crystallization [29].

**Figure 1:**
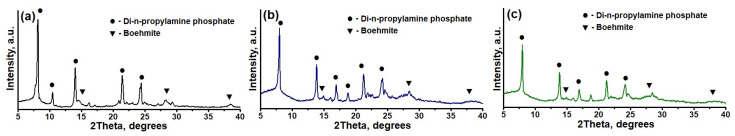
X-ray patterns of initial SAPO-gels prepared using various boehmites: (**a**) Sample SAPO-TR(20); (**b**) Sample SAPO-SB(20); (**c**) SAPO-RP(20).

On Figure 1 shows X-ray patterns of the dehydrated initial gels prepared using various boehmites, in Table 2 their phase composition. 

**Figure 2:**
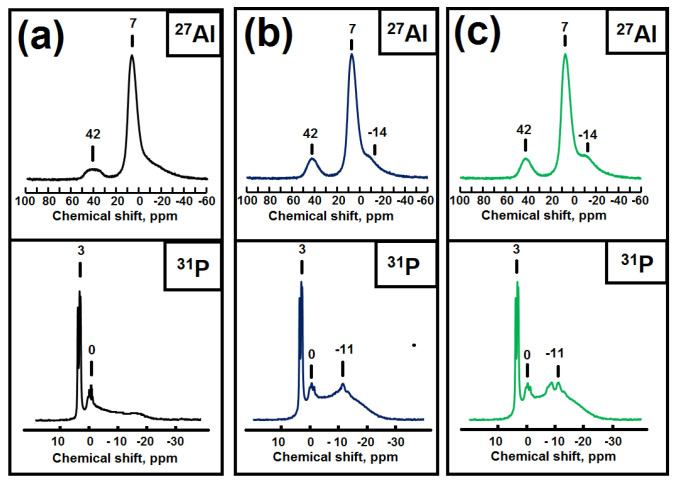
MAS NMR spectra of ^27^Al and ^31^P initial silicoaluminophosphate gels prepared using various boehmites: (**a**) Sample SAPO-TR(20); (**b**) Sample SAPO-SB(20); (**c**) SAPO-RP(20).

For a more detailed assessment of the interaction between the initial compounds during the preparation of gels, the MAS NMR spectra of ^27^Al and ^31^P were recorded (Figure 2). In the MAS NMR ^27^Al spectra for all samples, a strong signal is observed at 7 ppm, which can be attributed to the original boehmite (aluminum atoms surrounded by oxygen [AlO_6_]), a weak signal at −14 ppm. refers to amorphous aluminophosphate (aluminum atoms surrounded by [AlO_6_]), and a weak signal at 42 ppm refers to amorphous aluminophosphates (to Al atoms with a tetrahedral oxygen environment [AlO_4_]) [30,31,32]. In the MAS NMR ^31^P spectra for these silicoaluminophosphate gel samples, a group of signals at 3, 0, and –11 ppm is observed. The signal at 3 ppm mostly arises from phosphorus compounds in which the P atom is not bonded to Al atoms [30,31,32]. Based on XRD data (Figure 1), this signal corresponds to di-n-propylamine phosphate. The group of signals at 0 ppm was assigned to the phosphoric acid. A broad signal at −11 ppm is characteristic of aluminophosphates P(OAl)nOH, where n varies from 1 to 4 [30,31,32]. Thus, the analysis of the MAS NMR spectra of ^27^Al and ^31^P allows us to conclude that in the initial gels there is a partial interaction between boehmite and phosphoric acid with the formation of X-ray amorphous aluminophosphates for all samples that are not detected by XRD. It can also be seen from the obtained results that the proportion of signals in the MAS NMR spectra of ^27^Al and ^31^P, which is characteristic of amorphous aluminophosphate, differs for gels prepared using different boehmites. There is an increase in the proportion of amorphous aluminophosphate in the next series of aluminum sources TR > SB > RP, which indicates significant differences in their reactivity.

Differences in the reactivity of boehmites found good agreement with the pH data of the gels. In the series of boehmites RP > SB > TR, an increase in the pH of gels prepared with their use is observed (Table 2). The increase in the pH of the gels is due to the fact that more reactive boehmites interact better with phosphoric acid to form aluminophosphate, thereby increasing the final pH value of the gels.

To understand the influence of the properties of boehmites on the properties of the formed gels for them, their X-ray diffraction patterns were studied, on the basis of which the sizes of boehmite crystals were calculated, and their porous structure was additionally studied.

**Figure 3:**
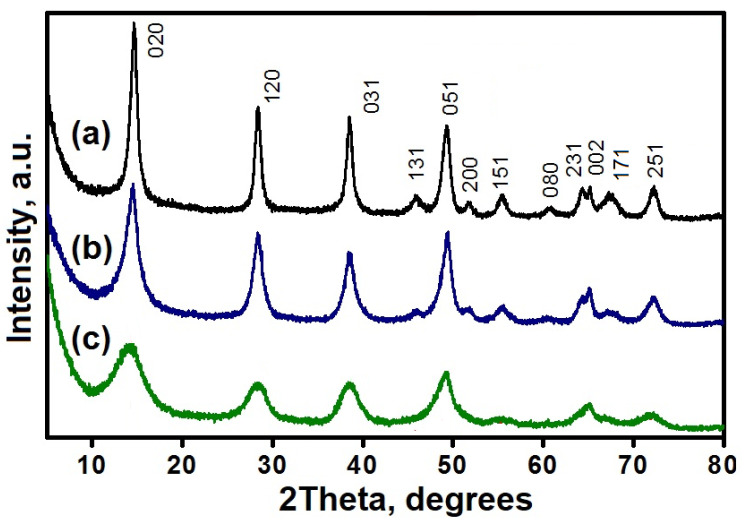
Boehmite X-ray patterns: (**a**) Sample TR KNT Group; (**b**) Sample Sasol SB; sample; (**c**) Sample PR KNT Group.

From Figure 3 it can be seen that the X-ray patterns of all samples correspond to the boehmite structure. The broadest peaks are observed for the RP sample, while the diffraction peaks are slightly narrower for the SB sample. The narrowest peaks are observed for the TR sample. This is due to the different sizes of crystallites. The smallest sizes of boehmite crystals are typical for the PR sample, the largest ones, for the TR sample.

In addition, for all boehmite samples, it can be seen that the (080) peak is much broader than the (002) peak. This is due to the fact that the average sizes of D001 crystallites in the [001] direction, i.e., in the layer plane is greater than the average dimensions D010 in the [010] direction, i.e., perpendicular to the layers. This can be explained by the fact that the chemical bonds in the layer are much stronger than between the layers. Table 3 presents the results of calculations of the size of boehmite crystals. It is also seen that the smaller the crystallite size, the greater the interplanar spacing d002 (or the spacing between layers).

Differences in crystal sizes have an important effect on the porous structure of boehmites. The PR sample has the highest specific surface area and mesopore volume, while the TR sample has the lowest indicated characteristics.

Thus, we see that the differences in the reactivity of boehmites are primarily due to the size of their crystals. The RP boehmite sample, due to its smaller crystals and more developed surface, interacts much better with phosphoric acid, forming amorphous aluminophosphate, compared to the TR and SB boehmite samples, which are characterized by larger crystals.

**Figure 4:**
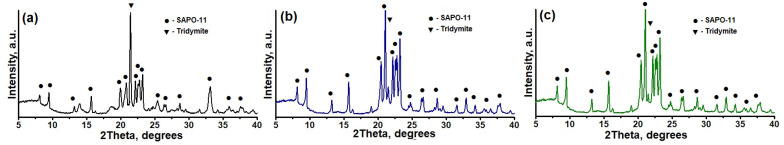
X-ray diffraction patterns of the crystallization products of the initial gels prepared using various boehmites: (**a**) Sample SAPO-11-TR(20); (**b**) Sample SAPO-11-SB(20); (**c**) Sample SAPO-11-RP(20).

Figure 4 shows X-ray patterns of the crystallization products of the initial gels prepared using various boehmites, Table 4 shows their chemical and phase composition. 

**Figure 5:**
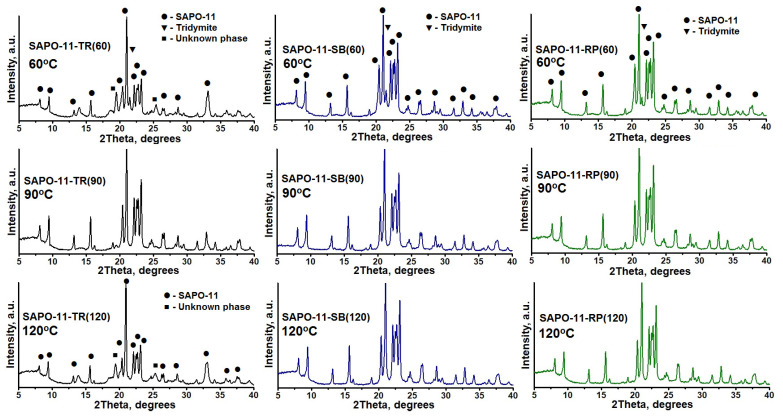
X-ray patterns of the crystallization products of the initial gels subjected to aging at a temperature in the range from 60 to 120 °C.

Previously, we showed [29] that for the synthesis of high-phase purity SAPO-11 using boehmite as a source of aluminum, it is necessary to carry out the aging stage at higher temperatures in order to form aluminophosphates containing ≡Al-O-P≡ bonds, which facilitates the crystallization process. Figure 5 shows X-ray patterns of the crystallization products of the initial gels subjected to aging at temperatures from 60 to 120 °C. It can be seen that the crystallization of gels subjected to a preliminary stage of aging at 60 °C leads to the formation of SAPO-11 with tridymite impurities, regardless of the aluminum source used. Crystallization of gels prepared using SB and RP boehmites allows obtaining high phase purity SAPO-11 only from systems aged at 90 to 120 °C. Crystallization of gels prepared with TR boehmite allows obtaining SAPO-11 of high phase purity only by subjecting it to aging at 90 °C, and higher temperatures led to the formation of a co-crystallized SAPO-11 with an unknown phase. Previously, we showed [29] the use of its isopropoxide as a source of aluminum makes it possible to obtain SAPO-11 of high phase purity from the initial gels without the stage of their aging. However, as noted above, Al isopropoxide is a less preferred raw material than boehmite for the synthesis of SAPO-n molecular sieves. Thus, it can be seen that the introduction of the stage of gel aging in a certain temperature range makes it possible to obtain SAPO-11 of high phase purity, regardless of the properties of the boehmite used.

**Figure 6:**
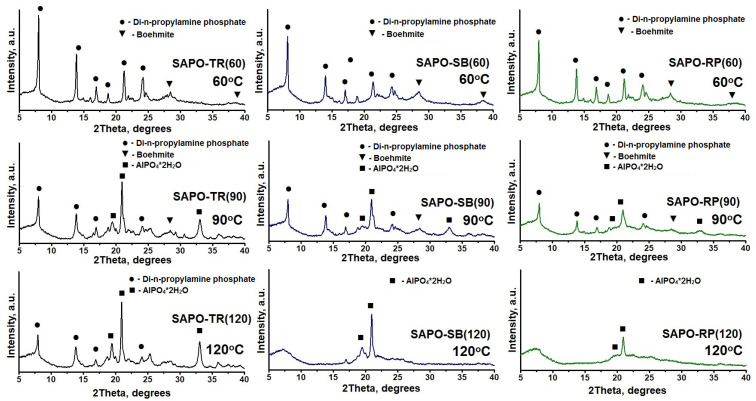
X-ray patterns of aged initial gels at 60 to 120 °C.

In order to understand the interconnections between boehmite properties, aging stage conditions, and crystallization results, aged gel samples were further examined by XRD, MAS NMR ^27^Al and ^31^P. Figure 6 shows X-ray patterns of aged gels at temperatures from 60 to 120 °C. It can be seen that all the gels that were aged at a temperature of 60 °C are a mixture of di-n-propylamine phosphate and unreacted boehmite phases, similar to the initial gels. Increasing the aging temperature to 90 °C for all gels leads to the formation of a mixture of phases consisting of crystalline hydroaluminophosphate AlPO_4_⋅2H_2_O (PDF no. 00-015-0311) and di-n-propylamine phosphate phases (PDF no. 00-039-1892) and unreacted boehmite (PDF No. 00-001-1283) contained in a modicum. With a further increase in the gel aging temperature to 120 °C, the AlPO_4_⋅2H_2_O phase becomes dominant.

**Figure 7:**
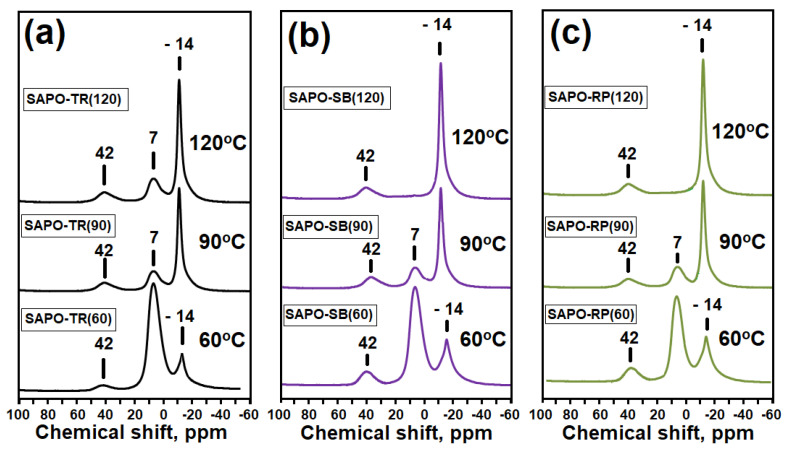
MAS NMR ^27^Al spectra of initial silicoaluminophosphate gels prepared using various boehmites and aged at 60–120 °C: (**a**) Samples of gels prepared using TR boehmite; (**b**) Gel samples prepared using boehmite SB; (**c**) Gel samples prepared using boehmite RP.

**Figure 8:**
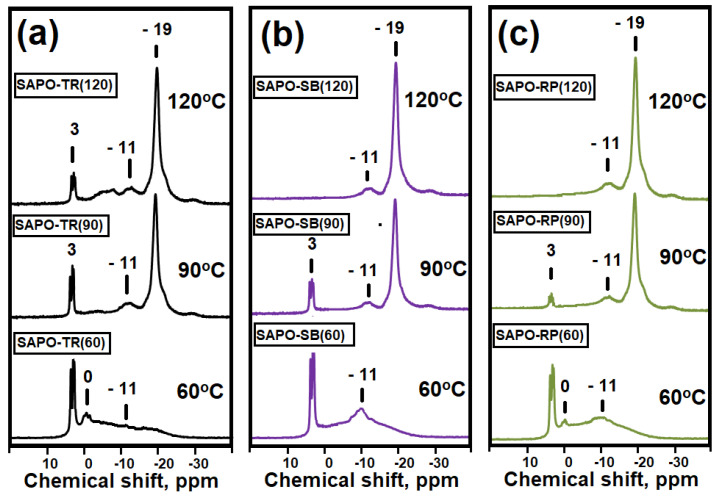
MAS NMR ^31^P spectra of initial silicoaluminophosphate gels prepared using various boehmites and aged at 60–120 °C: (**a**) Samples of gels prepared using TR boehmite; (**b**) Gel samples prepared using boehmite SB; (**c**) Gel samples prepared using boehmite RP.

Figure 7 and Figure 8 show the MAS NMR spectra of ^27^Al and ^31^P gel samples subjected to aging. The MAS NMR ^27^Al spectra show three main signals at −14, 7, and 42 ppm. The signals are observed at –14 ppm associated with [AlO_6_] in crystalline aluminophosphates. This signal can be attributed to the AlPO_2_ × 2H_2_O phase. The weak intensity bands at 7 ppm and 42 ppm are ascribed to the presence unreacted boehmite of and amorphous aluminophosphate, respectively. In the MAS NMR ^31^P spectra of samples, a band at −19 ppm is ascribed to [PO_4_] in crystalline aluminophosphates. Comparing the XRD and ^27^Al data, this signal is associated with AlPO_4_ × 2H_2_O. The bands at −11 ppm and −5 ppm are associated with aluminophosphates P(OAl)_n_OH, where n varies from 1 to 4, and a weak signal at 3 ppm, which is characteristic of di-n-propylamine phosphate. It can be seen that an increase in the gel aging temperature from 60 to 120 °C leads to an increase in the intensity in the MAS NMR spectra of ^27^Al signals at −14 ppm, and in the spectra of ^31^P at –19 ppm. For signals ^27^Al at (7, 42 ppm) and ^31^P at (−11, 0, 3 ppm), a decrease in intensity is observed. The results obtained indicate the predominant formation of the aluminophosphate phase as a result of the interaction between di-n-propylamine phosphate and the remaining boehmite. An increase in the gel aging temperature to 90 and 120 °C, as compared to 60 °C, leads to a deeper interaction of the gel components with the formation of aluminophosphates, among which the main phase is AlPO_4_* (H_2_O)_2_ crystalline hydroaluminophosphate. Comparison of the spectra of ^27^Al and ^31^P gels subjected to aging also shows that the properties of boehmite have a significant effect on the formation of the share of aluminophosphates. Thus, in the samples of gels obtained at 60 °C, an increase in the intensity of signals ^27^Al at (−14, 42 ppm) and ^31^P at (−11) is observed, and for gels obtained at 120 °C, a decrease in the intensity of signals ^27^Al at 7 ppm) in the series of TR, SB, and RP boehmites. The results obtained indicate that boehmite RP, due to the smallest crystal size, interacts better than other aluminum sources with phosphorus compounds with the formation of aluminophosphate phases, while boehmite TR, due to the largest crystals, does not fully react with phosphorus compounds even when the gel is aged at 120 °C.

Thus, comparing the materials obtained by aging of the initial gels, it can be seen that the use of sources of aluminum, the crystals of which have a more developed surface and have a smaller crystal size, leads to the formation of aluminophosphates, in which bonds of the ≡Al-O-P≡ type predominantly predominate. In addition, the synthesis of SAPO-11 of high phase purity is only possible in the case when an aluminophosphate of the AlPO_4_ × 2H_2_O type is present in the initial gel. The results obtained are due to the fact that bonds of the ≡Al-O-P≡ type have already been formed in aluminophosphates, which are future fragments of the SAPO-11 molecular sieve, which can significantly facilitate crystallization. However, it remains unclear why, in the case of the SAPO-TR(120) gel prepared using TR boehmite, the synthesis of SAPO-11 of high phase purity is impossible.

**Figure 9:**
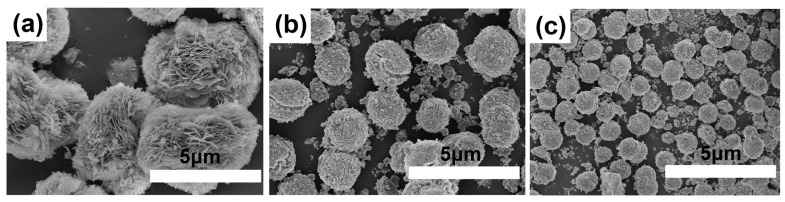
SEM images of gels aged at 120 °C: (**a**) Sample SAPO-TR(120); (**b**) Sample SAPO-SB(120); (**c**) Sample SAPO-RP(120).

On Figure 9 shows SEM images of gels aged at 120 °C, which are predominantly AlPO_4_ × 2H_2_O phases. It can be seen that the AlPO_4_ × 2H_2_O samples are spherical aggregates formed by thin plates. A decrease in the size of AlPO_4_ × 2H_2_O spherical aggregates is observed when using boehmites with a smaller crystal size. Apparently, large AlPO_4_ × 2H_2_O crystals obtained using TR boehmite do not have time to recrystallize into the SAPO-11 structure, as a result of which impurities of other crystalline silicoaluminophosphates are formed.

Thus, the results presented above show that the size of boehmite crystals as well as its porous structure can have a significant impact on the results of SAPO-11 crystallization.

**Figure 10:**
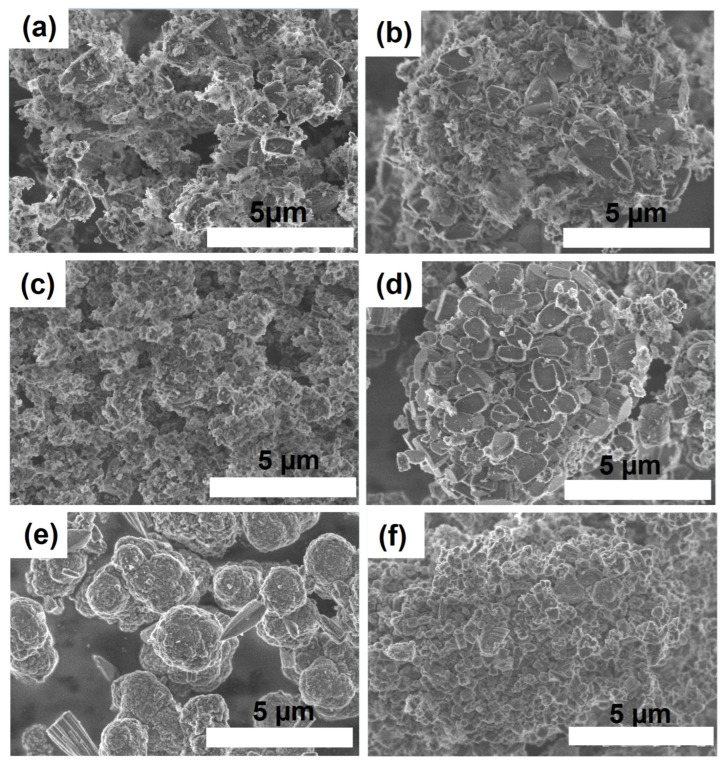
SEM images of crystallization products of silicoaluminophosphate gels prepared using various boehmites: (**a**) Sample SAPO-11-TR(60); (**b**) Sample SAPO-11-TR(90); (**c**) Sample SAPO-11-SB(90); (**d**)-Sample SAPO-11-SB(120); (**e**) Sample SAPO-11-PR(90); (**f**) Template SAPO-11-PR(120).

It is known that the morphology and dispersion of molecular sieves has a significant effect on their adsorption and catalytic properties. Figure 10 shows SEM images of SAPO-11 samples synthesized using various boehmites and aging temperatures of the initial gels. It can be seen that the sample (SAPO-11-SB(60)), obtained using TR boehmite, is a mixture of SAPO-11 crystals with a conical morphology of 1 to 2 µm in size and thin plates of tridymite with a size of 0.5 to 1 µm. Raising the aging temperature of the initial gel to 90 °C makes it possible to obtain SAPO-11 in the form of a mixture of cone-shaped crystals with a size of 1 to 2 µm and crystals in the form of prisms with a size of 0.2 to 0.3 µm. Upon transition to the more reactive boehmite Sasol SB, a significant change in the morphology of SAPO-11 is observed. The SAPO-11-SB(90) sample is characterized by nanocrystals of cubic morphology ranging in size from 100 to 300 nm. Raising the aging temperature to 120 °C (sample SAPO-11-SB(120)) already leads to the formation of crystals in the form of spherical intergrowths consisting of cones 0.5 to 1 µm in size. Crystallization of gels prepared using RP boehmite leads to the formation of SAPO-11 in the form of spherical aggregates with a size of 1 to 2 μm, formed from nanocrystals. A further increase in the aging temperature of such a gel to 120 °C also makes it possible to obtain cubic nanocrystals with sizes from 200 to 500 nm. It should be noted that the use of its isopropoxide as a source of Al leads to the formation of large aggregates of SAPO-11 crystals 6 to 8 μm in size [29]. In such intergrowths, the inner part of the crystals may be inaccessible to the reactant molecules. The use of boehmites, on the contrary, makes it possible to obtain individual nanocrystals, for which more efficient diffusion is ensured.

**Figure 11:**
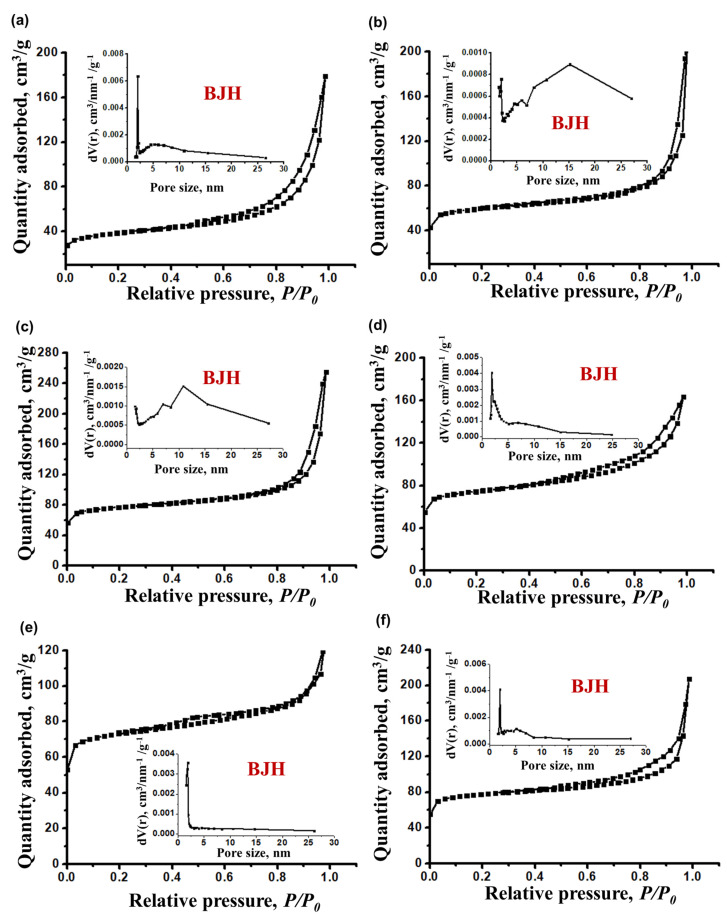
Nitrogen adsorption-desorption isotherms and pore size distribution of silicoaluminophosphate molecular sieves: (**a**) Sample SAPO-11TR(60); (**b**) Sample SAPO-11TR(90); (**c**) Sample SAPO-11SB(90); (**d**) Sample SAPO-11SB(120); (**e**) Sample SAPO-11RP(90); (**f**) Sample SAPO-11RP(120).

Differences in the dispersity and morphology of crystals have a significant effect on the porous structure of SAPO-11. Figure 11 shows N_2_ adsorption–desorption isotherms, pore size distribution, and Table 5 shows the characteristics of the porous structure of samples of crystalline silicoaluminophosphates. It can be seen that for all samples of molecular sieves, isotherms type II with a hysteresis loop, which are characteristic of micro-mesoporous materials, are observed [33]. These samples are characterized by a wide distribution of mesopores in size from 2 to 25 nm, which are formed in the intercrystalline space (Figure 10). Samples SAPO-11SB(90) and SAPO-11RP(120) are characterized by the greatest mesopore volume and specific surface area. The results obtained are explained by the fact that their secondary porosity is voids between nanocrystals ranging in size from 1 × 10^2^ to 5 × 10^2^ nm. Such a micro-mesoporous structure is usually called hierarchical. It is important to note that SAPO-11 hierarchical samples were obtained by a relatively simple method without the use of pore-forming templates and crystal growth modifiers, which makes it possible to obtain these materials in a relatively affordable and economically viable way [34]. The SAPO-11TR(60) sample is characterized by the smallest specific surface area and micropore volume, which is due to the presence of non-porous tridymite in its composition.

Thus, using aluminum sources with different crystal sizes in the synthesis of SAPO-11 molecular sieves, as well as changing the conditions of the aging stage of the initial gels, it is possible to control the fineness and morphology, as well as the characteristics of the secondary porous structure of SAPO-11 silicoaluminophosphate molecular sieves. It is important to note that SAPO-11 hierarchical molecular sieves were synthesized without the use of crystal growth modifiers and pore-forming templates.

As noted above, SAPO-11 silicoaluminophosphate molecular sieves are characterized by the presence of “moderate” acidic sites and a one-dimensional channel porous structure. The symbiosis of these phenomena leads to the appearance of special catalytic properties of silicoaluminophosphates.

**Figure 12:**
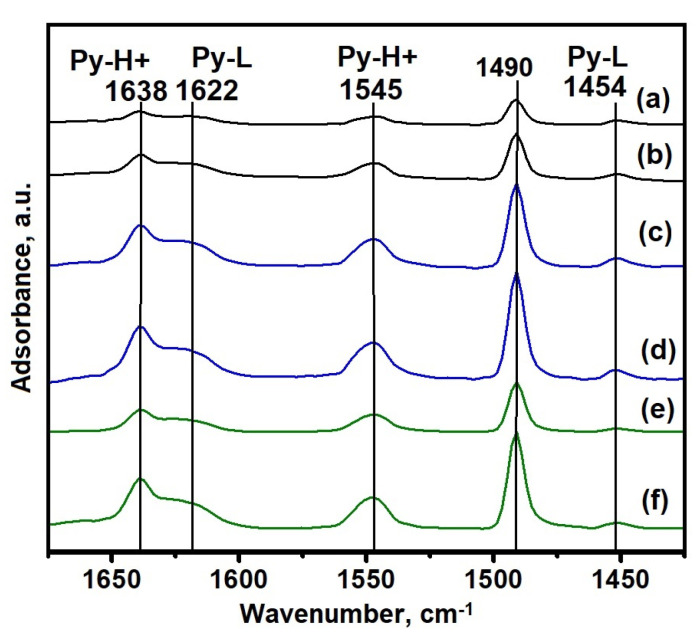
IR spectra of adsorbed pyridine for SAPO-11: (**a**) Sample SAPO-11TR(60); (**b**) Sample SAPO-11TR(90); (**c**) Sample SAPO-11SB(90); (**d**) Sample SAPO-11SB(120); (**e**) Sample SAPO-11RP(90); (**f**) Sample SAPO-11RP(120).

Figure 12 shows the IR spectra of adsorbed pyridine, and Table 6 shows the calculated concentrations of acid sites in SAPO-11 samples after crystallization. It can be seen that absorption bands (abs) at 1545 and 1455 cm^−1^ are observed for all samples, which are related to pyridine adsorbed on Brønsted and Lewis acid sites, respectively [35]. The absorption band at 1490 cm^−1^ is usually attributed to pyridine adsorbed on both types of molecular sieve sites. For samples containing tridymite, lower concentrations of acid sites are observed because tridymite is a non-porous material. It should be noted that the samples (SAPO-11-SB(90), SAPO-11-RP(120)) with a hierarchical porous structure are characterized by a higher concentration of acid sites, despite the close content of silicon. The results obtained are due to a more developed secondary porous structure in these samples, which provides more efficient access of pyridine molecules to acid sites. Samples SAPO-11-SB(90) and SAPO-11-RP (120) are characterized by a similar content of silicon; however, the concentration of acid sites in the first sample is higher due to the better accessibility of the latter due to a more developed mesoporous structure.

**Figure 13:**
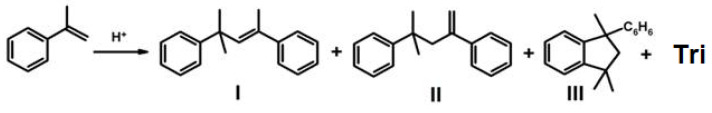
Reaction oligomerization of α-methylstyrene.

It was shown in [36] that the SAPO-11-based catalyst is a highly selective system for the production of linear dimers of α-methylstyrene (Figure 13). Oligomerization of α-methylstyrene is a practically important process. Linear dimers of α-methylstyrene is a suitable for uses as a polymer chain growth regulators. Linear dimers of α-methylstyrene is used in the production of polymers and rubbers, as a plasticizers, and for production dielectric liquids and synthetic lubricants. It was previously shown [37] that in the presence of zeolite-containing catalysts, α-methylstyrene oligomerization proceeds with the formation of linear (I, II) and cyclic (III) dimers, as well as trimers (Tri).

The results of dimerization process of α-methylstyrene on SAPO-11 synthesized using different boehmites are shown in Table 7. It can be seen that on all SAPO-11 samples, the main reaction products are α-methylstyrene dimers of linear (I, II) and cyclic (III) structures, and the formation of trimers (Tri) is also observed. Among the dimers, product II is the main one. It is important to note that, in view of the bulk structure of the dimerization products, the reaction most likely occurs near the mouths of pores. Comparison of the results of dimerization on different samples shows that samples containing tridymite exhibit lower activity, which is due to their porous structure, in which the volume of mesopores is significantly less. The most active sample is SAPO-11-SB(90), which is characterized not only by the highest concentration of acid sites, but also by a more developed secondary porous structure, which provides more efficient access to acid sites. For SAPO-11-SB(90) and SAPO-11-RP(120) samples, similar conversions of α -methylstyrene and selectivity for linear dimers are observed. The results obtained are due to close values of the concentrations of acid sites and characteristics of the porous structure.

Thus, the obtained results show that for SAPO-11, using boehmites with different properties as a source of aluminum and changing the conditions of the aging stage of the initial gels, it is possible to control the characteristics of the secondary porous structure, morphology, and acidity of materials. This feature makes it possible to synthesize SAPO-11 samples providing the best performance in catalytic transformations at similar silicon contents in the initial reaction gels.

## 3. Conclusions

The formation of silicoaluminophosphate gels with the composition Al boehmite source with different properties.

It is shown that the size of crystals and the porous structure of boehmite have a significant effect on the chemical and phase properties of the formed gels and the products of their subsequent crystallization. It has been established that boehmites with smaller crystals and a more developed porous structure interact better with phosphoric acid at the initial stages of gel preparation, thus forming amorphous aluminophosphate. Crystallization of gels containing a large proportion of amorphous aluminophosphate makes it possible to reduce the proportion of tridymite in their crystallization products. It was found that without carrying out a preliminary stage of aging of the initial gel in the temperature range from 90 to 120 °C, it is impossible to obtain SAPO-11 of high phase purity, because the formation of non-porous tridymite and cristabolite is observed.

Methods for controlling the morphology and secondary porous structure of SAPO-11 are proposed based on the use of boehmites with different crystal sizes and gel aging stage conditions at temperatures from 90 to 120 °C.

A method for the synthesis of SAPO-11 with a hierarchical porous structure (SBET = 250 m^2^/g, Vmeso = 0.32 cm^3^/g) formed from nanocrystals 200 to 400 nm in size was proposed.

The resulting SAPO-11 samples were studied in the dimerization reaction of α-methylstyrene. It is shown that the maximum values of monomer conversion and selectivity for linear dimers are observed when using a sample of silicoaluminophosphate with a hierarchical porous structure.

## 4. Materials and Methods

### 4.1. Preparation of Silicoaluminophosphate Gels

Silicoaluminophosphate gels were prepared using phosphoric acid (H_3_PO_4_, 85%, Reachim, Moscow, Russia), boehmites ((SB, AlO(OH), 78% Al_2_O_3_, Sasol SB, Hamburg, Germany), (TR, AlO(OH), 82 % Al_2_O_3_, KNT Group, Ishimbay, Russia), (RP, AlO(OH), 72% Al_2_O_3_, KNT Group, Ishimbay, Russia)) silica sol obtained by the sol-gel method [34], and di-n-propylamine ( DPA, 99%, Acros Organics, Schwerte, Germany). The molar compositions of the synthesis gel mixtures were 1.0Al_2_O_3_•1.0P_2_O_5_•0.3SiO_2_•1.0DPA•40H_2_O. A general synthesis procedure was as follows. The reaction mixture preparation consisted of the following steps: (i) At room temperature 24.0 g of distilled water, boehmite (5.6 g PB, 5.1 g TR, 6.2 g RP), and 4.4 g di-n-propylamine were successively added to 10.0 g of phosphoric acid and stirred intensively for 1 h. Then, the calculated amount of silica sol was poured into the resulting gel and intensively stirred for 1 h, then subjected to aging a thermostat in the temperature range from 20 to 120 °C for 24 h.

### 4.2. Crystallization of Silicoaluminophosphate Molecular Sieves

SAPO-11 molecular sieves were crystallized from the appropriate silicoaluminophosphate gels at temperatures ranging from 190 to 210 °C for twenty-four hours. Preliminary experiments have shown that holding crystallization for more than twenty-four hours leads to the formation of nonporous cristobalite. Table 1 also lists the conventions for SAPO-11 samples prepared from the respective silicoaluminophosphate gels.

### 4.3. Characterization

Energy dispersive X-ray fluorescence spectrometer Shimadzu EDX-7000P were used of the elemental composition of the samples initial silicoaluminophosphate gels and their crystallization products by method of fundamental parameters.

X-ray diffraction (XRD) spectra of non-calcined SAPO-11 samples were collected by Shimadzu XRD-7000 diffractometer using Cu Kα radiation. Scanning was carried out in the range of 2θ angles from 5 to 40° with a step of 1 deg/min. The phase analysis of the obtained X-ray patterns was carried out using the PDF2 database. The degree of crystallinity was estimated from the intensity of the amorphous halo in the region from 20 to 30° 2θ.

^31^P and ^27^Al NMR spectra have been acquired on a Bruker Avance-II 400 WB spectrometer using a 4 mm H/X MAS WVT probe with operating frequencies of 162.0 and 104.2 MHz, respectively, and frequency VMU—12 kHz. To obtain the MAS NMR spectra on ^31^P nuclei, a single-pulse technique (90-degree pulse) was used with the following recording parameters: pulse duration, 2.1 μs; the number of repetitions—32; time between repetitions—120 s. The MAS NMR spectra on ^27^Al nuclei were obtained using a 15-degree excitation pulse with a duration of 0.8 μs; the number of repetitions—256; the time between repetitions is 0.5 s.

The size and morphology of the SAPO-11 crystals were determined by emission scanning electron microscopy (SEM). SEM imaging was performed on Hitachi Regulus SU8220 operating at 5 kV.

The volume of micro- and mesopores on the BET surface area were determined by nitrogen physisorption measurements using Quantachrome Nova 1200e sorbtometer. The specific surface area was calculated using the multipoint BET method. The volume of micropores in the presence of mesopores was estimated by the t-Plot method. The pore size distribution was calculated using the BJH model (Halenda) along the desorption branch.

The IR-spectra of adsorbed pyridine were recorded on a Bruker Vertex-70V IR Fourier spectrometer. The diameter of the tablet for recording IR spectra was 0.1 cm. The spectra were recorded with a resolution of 4 cm^–1^ in the range from 4 × 10^2^ to 4 × 10^3^ cm^−1.^ The adsorption of pyridine was carried out at 423 K for 30 min, then the physisorbed pyridine was removed by evacuation at 423 K for 30 min. Lewis acid sites (LAS) at 1454 cm^−1,^ and Brønsted acid sites (BAS) were quantitatively assessed by integrating the peak at 1545 cm^−1^, based on the integral molar extinction coefficients of pyridine known in the literature for centers of each type [35].

### 4.4. Method for Studying the Catalytic Properties of Samples in the Dimerization Process of α-Methylstyrene

The catalytic properties of SAPO-based catalyst in the dimerization of α-methylstyrene were studied in an isothermal batch reactor with constant intensive stirring. Reaction temperature was 25 °C; the other test conditions were as follows: a pressure of 0.1 MPa, and a chlorobenzene/α-methylstyrene (99%, Acros Organics) mass ratio of 4, reaction time varied in a range from 2 to 6 h, the weight concentration catalyst/α-methylstyrene was 10%. Prior to testing, the catalyst (80–100 μm fraction of the calcined zeolite) was kept in a flow of dried helium at 350 °C from 3 to 4 h before the reaction. The reaction products were subjected to chromatographic analysis using a HRGS 5300 Mega Series “Carlo Erba” chromatograph with a flame ionization detector (the glass capillary column of 25 length, the SE-30 phase, temperature of analysis varied in a range from 50 to 280 °C, programmed heating of 8 °C min^−1^, detector temperature of 250 °C, evaporator temperature of 300 °C, flow rate of helium carrier gas of 30 mL/min).

## Figures and Tables

**Table 1:** Explanation of symbols silicoaluminophosphate gels and crystallization products based on them.

Boehmite	Gel Aging Temperature, °C	Gel Sample	Sample SAPO-11
TR	20	SAPO-TR(20)	SAPO-11-TR(20)
TR	60	SAPO-TR(60)	SAPO-11-TR(60)
TR	90	SAPO-TR(90)	SAPO-11-TR(90)
TR	120	SAPO-TR(120)	SAPO-11-TR(120)
SB	20	SAPO-SB(20)	SAPO-11-SB(20)
SB	60	SAPO-SB(60)	SAPO-11-SB(60)
SB	90	SAPO-SB(90)	SAPO-11-SB(90)
SB	120	SAPO-SB(120)	SAPO-11-SB(120)
RP	20	SAPO-RP(20)	SAPO-11-RP(20)
RP	60	SAPO-RP(60)	SAPO-11-RP(60)
RP	90	SAPO-RP(90)	SAPO-11-RP(90)
RP	120	SAPO-RP(120)	SAPO-11-RP(120)

**Table 2:** Chemical and phase composition of the SAPO-gels.

Sample	Gel Al_2_O_3_●P_2_O_5_●SiO_2_	pH Index	Phase Composition
SAPO-TR(20)	1.00●1.01●0.31	2.0	Ph.DPA ^1^ + Bh ^2^
SAPO-TR(60)	1.00●0.98●0.32	2.4	Ph.DPA + Bh
SAPO-TR(90)	1.00●1.02●0.29	4.3	Ph.DPA + AlPO_4_ × H_2_O
SAPO-TR(120)	1.00●1.00●0.30	6.6	AlPO_4_ × 2H_2_O
SAPO-SB(20)	1.00●1.03●0.29	3.0	Ph.DPA + Bh
SAPO-SB(60)	1.00●1.02●0.30	3.5	Ph.DPA + Bh
SAPO-SB(90)	1.00●1.03●0.29	5.9	Ph.DPA + AlPO_4_ × 2H_2_O
SAPO-SB(120)	1.00●1.01●0.31	7.3	AlPO_4_ × 2H_2_O
SAPO-RP(20)	1.00●1.02●0.30	3.2	Ph.DPA + Bh
SAPO-RP(60)	1.00●0.99●0.31	4.3	Ph.DPA + Bh
SAPO-RP(90)	1.00●1.01●0.30	6.2	Ph.DPA + AlPO_4_ × 2H_2_O
SAPO-RP(120)	1.00●0.98●0.30	7.8	AlPO_4_ × 2H_2_O

^1^—Di-n-propylamine phosphate; ^2^—Boehmit.

**Table 3:** Crystal sizes and porous structure of boehmites.

Sample	Crystal Sizes			Porous Structure	
	D_001_^1^, nm	D_010_^2^, nm	d_020_^3^, Å	S_BET_^4^, m^2^/g	V_meso_^5^, cm^3^/g
TR	21	11	6.05	152	0.54
SB	16	6	6.14	236	0.64
PR	13	3	6.25	340	0.88

^1^—Average sizes of crystallites in the plane of layers; ^2^—Average dimensions of crystallites perpendicular to the plane of the layers; ^3^—Interplanar distances (distances between layers); ^4^—Specific surface according to BET; ^5^—Specific volume of mesopores.

**Table 4:** Degree of crystallinity, chemical and phase composition of crystallization products of silicoaluminophosphate gels.

Sample	Gel Al_2_O_3_●P_2_O_5_●SiO_2_	Phase Composition	DR ^1^, %
SAPO-TR(20)	1.00●1.00●0.13	SAPO-11 + Tr ^2^	-
SAPO-TR(60)	0.00●0.97●0.15	SAPO-11 + Tr	-
SAPO-TR(90)	0.00●0.95●0.18	SAPO-11	85
SAPO-TR(120)	1.00●0.96●0.17	SAPO-11 + Unknown phase	-
SAPO-SB(20)	1.00●0.99●0.14	SAPO-11 + Tr	-
SAPO-SB(60)	0.00●0.98●0.17	SAPO-11 + Tr	-
SAPO-SB(90)	0.00●0.96●0.22	SAPO-11	95
SAPO-SB(120)	1.00●0.97●0.19	SAPO-11	97
SAPO-RP(20)	1.00●0.99●0.15	SAPO-11 + Tr	-
SAPO-RP(60)	0.00●0.97●0.16	SAPO-11 + Tr	-
SAPO-RP(90)	0.00●0.95●0.20	SAPO-11	94
SAPO-RP(120)	1.00●0.96●0.21	SAPO-11	96

^1^—Degree of crystallinity; ^2^—Tridymite.

**Table 5:** Characteristics of the porous structure of molecular sieves.

Sample	S_BET_^1^, m^2^/g	S_EX_^2^, m^2^/g	V_micro_^3^, cm^3^/g	V_meso_^4^, cm^3^/g
SAPO-11TR(60)	128	76	0.03	0.25
SAPO-11TR(90)	192	94	0.05	0.30
SAPO-11SB(90)	250	118	0.07	0.32
SAPO-11SB(120)	240	113	0.07	0.18
SAPO-11RP(90)	233	103	0.07	0.13
SAPO-11RP(120)	247	117	0.07	0.25

^1^—specific surface according to BET. ^2^—external specific surface. ^3^—specific volume of micropores. ^4^—specific volume of mesopores.

**Table 6:** Acid Site Concentrations of SAPO-11 Silicoaluminophosphates According to IR Spectroscopy with Pyridine Adsorption.

Sample	Acidity (μmol/g)		
	BAS ^1^	LAS ^2^	Ʃ BAS+LAS ^3^
SAPO-11TR(60)	17	5	22
SAPO-11TR(90)	37	7	44
SAPO-11SB(90)	75	10	85
SAPO-11SB(120)	97	12	109
SAPO-11RP(90)	50	4	54
SAPO-11RP(120)	88	7	95

^1^—Bronsted Acid Sites. ^2^—Lewis Acid Sites. ^3^—Total Acid Site Concentration.

**Table 7:** Oligomerization of α-methylstyrene over SAPO-11 molecular sieves.

Sample	X_a-MS_^1^, %	S_I_^2^, %	S_II_^3^, %	S_III_^4^, %	S_Tri_^5^, %
SAPO-11TR(60)	36.1	2.1	95.1	2.7	0.1
SAPO-11TR(90)	44.5	2.6	94.2	3.0	0.2
SAPO-11SB(90)	92.4	4.5	90.7	4.2	0.6
SAPO-11SB(120)	75.2	3.8	92.2	3.5	0.5
SAPO-11RP(90)	62.1	2.8	93.3	3.6	0.3
SAPO-11RP(120)	88.0	4.6	91.2	3.7	0.5

^1^—Conversion of a-methylstyrene. ^2^—Linear dimer selectivity. ^3^—Linear Dimer Selectivity. ^4^—Selectivity for cyclic dimer III. ^5^—Selectivity for trimers.

## Data Availability

Not applicable.
